# *JrGA20ox1*-transformed rootstocks deliver drought response signals to wild-type scions in grafted walnut

**DOI:** 10.1093/hr/uhae143

**Published:** 2024-05-24

**Authors:** Heqiang Lou, Fengmin Wang, Jiaqi Zhang, Guangli Wei, Jingjing Wei, Hengkang Hu, Yan Li, Ketao Wang, Zhengjia Wang, Youjun Huang, Jiasheng Wu, Dong Pei, Jianqin Huang, Qixiang Zhang

**Affiliations:** State Key Laboratory of Subtropical Silviculture, Zhejiang A&F University, Hangzhou, Zhejiang 311300, China; State Key Laboratory of Subtropical Silviculture, Zhejiang A&F University, Hangzhou, Zhejiang 311300, China; Zhejiang Provincial Key Laboratory of Forest Aromatic Plants-based Healthcare Functions, Zhejiang A&F University, Hangzhou, Zhejiang 311300, China; State Key Laboratory of Subtropical Silviculture, Zhejiang A&F University, Hangzhou, Zhejiang 311300, China; State Key Laboratory of Subtropical Silviculture, Zhejiang A&F University, Hangzhou, Zhejiang 311300, China; State Key Laboratory of Subtropical Silviculture, Zhejiang A&F University, Hangzhou, Zhejiang 311300, China; State Key Laboratory of Subtropical Silviculture, Zhejiang A&F University, Hangzhou, Zhejiang 311300, China; State Key Laboratory of Subtropical Silviculture, Zhejiang A&F University, Hangzhou, Zhejiang 311300, China; State Key Laboratory of Subtropical Silviculture, Zhejiang A&F University, Hangzhou, Zhejiang 311300, China; State Key Laboratory of Subtropical Silviculture, Zhejiang A&F University, Hangzhou, Zhejiang 311300, China; State Key Laboratory of Subtropical Silviculture, Zhejiang A&F University, Hangzhou, Zhejiang 311300, China; Zhejiang Provincial Key Laboratory of Forest Aromatic Plants-based Healthcare Functions, Zhejiang A&F University, Hangzhou, Zhejiang 311300, China; State Key Laboratory of Subtropical Silviculture, Zhejiang A&F University, Hangzhou, Zhejiang 311300, China; State Key Laboratory of Tree Genetics and Breeding, Key Laboratory of Tree Breeding and Cultivation of State Forestry and Grassland Administration, Research Institute of Forestry, Chinese Academy of Forestry, Beijing 100091, China; State Key Laboratory of Subtropical Silviculture, Zhejiang A&F University, Hangzhou, Zhejiang 311300, China; State Key Laboratory of Subtropical Silviculture, Zhejiang A&F University, Hangzhou, Zhejiang 311300, China; Zhejiang Provincial Key Laboratory of Forest Aromatic Plants-based Healthcare Functions, Zhejiang A&F University, Hangzhou, Zhejiang 311300, China

## Abstract

Targeted regulation using transgrafting technology has become a trend. However, the mechanisms of transgene-derived signal communication between rootstocks and scions remain unclear in woody plants. Here, we grafted wild-type (WT) walnut (*Juglans regia* L.) on WT (WT/WT), *JrGA20ox1* (encodes a gibberellin 20-oxidase)-overexpressing (WT/OE), and *JrGA20ox1*-RNAi transformation (WT/RNAi) walnut *in vitro*. We aimed to elucidate the mechanisms of *JrGA20ox1*-derived signal communication under PEG-simulated drought stress between rootstocks and scions in walnut. We demonstrated that *JrGA20ox1*-OE and *JrGA20ox1*-RNAi rootstocks could transport active gibberellins (GAs) and *JrGA20ox1*-RNAi vector-produced sRNAs to WT scions under PEG-simulated drought stress, respectively. The movement of sRNAs further led to a successive decline in *JrGA20ox1* expression and active GA content. Meanwhile, unknown mobile signals may move between rootstocks and scions. These mobile signals reduced the expression of a series of GA-responsive and GA-non-responsive genes, and induced ROS production in guard cells and an increase in ABA content, which may contribute to the drought tolerance of WT/RNAi, while the opposite occurred in WT/OE. The findings suggest that *JrGA20ox1*-derived rootstock-to-scion movement of signals is involved in drought tolerance of scions. Our research will provide a feasible approach for studying signal communication in woody plants.

## Introduction

Grafting originated 3000 ago and has been utilized for various purposes in woody stock/scion plants, including asexual propagation, enhancing precocity and yields, domesticating new woody crops, shortening juvenile periods, creating dwarf trees for easier harvesting, and providing abiotic and biotic resistance [1–6]. The phenotypes of grafted scions vary depending on the rootstock partners [5]. Increasing evidence suggests that various constituents, such as ions, hormones, peptides/proteins, and nucleic acids (including small RNAs), can move between scions and rootstocks bidirectionally [7, 8]. These mobile constituents from one graft partner have the potential to induce phenotypic changes in the other graft partner [5].

The management of specific signals moving from rootstock to scion is of great interest, particularly with the integration of transgenic technologies. This has led to the rise in popularity of transgrafting, a grafting strategy involving the combination of a non-transformed scion with a transformed rootstock, or vice versa [9]. The main goal of transgrafting is to enhance the food security of biotech crops by ensuring that the fruits harvested from a non-transformed scion remain genetically unmodified [10].

Transgrafting technology has been successfully utilized in various fruit trees, including apple (*Malus* × *domestica*) [11], sweet cherry (*Prunus avium* L.) [12, 13], plum (*Prunus domestica* var. ‘Stanley’) [14], walnut (*Juglans regia* L.) [15], and blueberry (*Vaccinium corymbosum* L.) [16]. The rootstock-to-scion movement of small RNAs, proteins, and phytohormones produced by transgenes was involved in disease resistance, tolerance to viral infection, regulation of plant size and morphology [17], etc. There has been a growing trend towards employing targeted regulation through transgrafting technology, although the mechanisms regulating the transmission of transgene-derived signals are not fully understood.

Efforts to detect the movement of transgene-derived signals between rootstocks and scions in transgrafted woody plants have shown varying results. Transformed apple rootstock expressing *RolB* was unable to translocate *RolB* transcripts into wild-type (WT) scion [18]. No translocation of *Gastrodia* antifungal protein (GAFP-1) from transformed rootstock to WT scion was detected in plum [14], while it has been demonstrated that transgene signal was transported from transformed rootstock to WT scions in apple and walnut [15, 19]. Interestingly, *PbWoxT1* mRNA can be transported from *PbWoxT1*-overexpressing tobacco (*Nicotiana tabacum*) rootstocks to WT scions, and this transport can be promoted when WT scions are grafted onto *PbWoxT1*- and *PbPTB3*-co-overexpressing tobacco rootstocks [20]. These inconsistencies in the mobility of transformed components across different studies suggest the complexity of transgene-derived signal trafficking, or they may be due to limitations in experimental systems for large trees. As a result, accurately predicting the actual effect of planned transgrafting remains challenging, and further research using feasible experimental methods is necessary.

*In vitro* grafting is a relatively new technique that combines the advantages obtained from conventional grafting with those of micropropagation [21]. Because it is based on micropropagation, *in vitro* grafting can be performed all year round and allows easy cultivation, ensuring the consistency and accuracy of experiments with woody plants. It has been applied successfully on some fruit and nut crops, including apple [19] and walnut [15]. Therefore, *in vitro* grafting combined with transgrafting must be a powerful strategy for studying the mechanisms of signal communication between rootstocks and scions in grafted woody plants.

Walnuts (*Juglans regia* L.) are the third most important nut crop in terms of world trade, and rank second in production behind cashews [22]. Like other tall economic trees, walnut is often difficult to harvest and often encounters drought stress, which is due to its higher stems and high water requirement [23]. Previous studies have revealed that gibberellin (GA) is involved in regulating a variety of growth processes and stresses, including both stem elongation and drought tolerance [24–27]. Among the 136 identified GAs, GA_1_, GA_3_, GA_4_, and GA_7_ show bioactivity [28]. It is noted that GA20-oxidases (GA20oxs) are key enzymes functioning in converting GA precursors to the active GA forms GA_1_, GA_3_, and GA_4_ [29]. Overexpression of *GA20ox* has been reported to increase plant height [30]. *Arabidopsis ga20ox1*/*2*, and *ga20ox1*/*2*/*3* mutants have reduced GA levels and display drought resistance [31]. In poplar, downregulation of PagGA20ox1 resulted in architecture change and could improve drought resistance by attenuating active GA synthesis [27]. Drought reduced GA accumulation by downregulating *GA20ox1* and *GA20ox2*, and mutations in these genes decreased water loss in tomato [26].

To gain both semi-dwarf and drought-tolerant trees and precisely elucidate the mechanisms of signal communication between rootstocks and scions in transgrafted woody plants, we generated transformed walnuts with overexpression of *JrGA20ox1*-GFP (*JrGA20ox1*-OE) and hairpin (hp) RNAi constructs containing self-complementary intron-spliced fragments of the *JrGA20ox1* gene sequence (*JrGA20ox1*-RNAi). Then, we conducted grafting experiments by *in vitro* grafting combined with transformation. WT scions were grafted onto WT (WT/WT), *JrGA20ox1*-OE (WT/OE), and *JrGA20ox1*-RNAi (WT/RNAi) rootstocks. We demonstrated that *JrGA20ox1*-OE and *JrGA20ox1*-RNAi could change the drought tolerance and growth of walnut in an opposite manner in non-grafted plants as well as in WT scions from transgrafted plants. We also revealed that the rootstocks from *JrGA20ox1*-OE and *JrGA20ox1*-RNAi could transport active GAs and small RNAs but not *JrGA20ox1* mRNAs to WT scions under PEG-simulated drought stress, and induced a series of responses to PEG simulated drought stress.

## Results

### *JrGA20ox1* positively regulates walnut shoot growth

**Figure 1:**
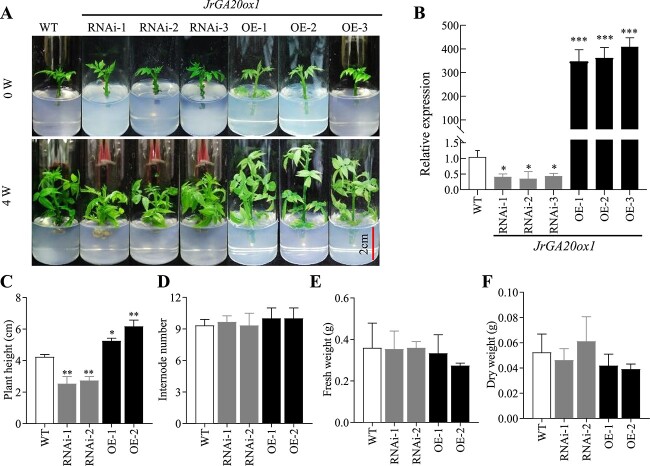
*JrGA20ox1* positively regulates walnut shoot growth. **A** Growth phenotypes of *JrGA20ox1*-RNAi and *JrGA20ox1*-OE plants. **B** qRT–PCR analysis of *JrGA20ox1* expression in WT, *JrGA20ox1*-RNAi, and *JrGA20ox1*-OE plants. **C**–**F** Plant height (**C**), internode number (**D**), fresh weight (**E**), and dry weight (**F**) of 2-week-cultured WT, *JrGA20ox1*-RNAi, and *JrGA20ox1*-OE plants. Statistically significant differences in comparison with WT are indicated (^*^P < 0.05, ^*^^*^P < 0.05, ^*^^*^^*^P < 0.001). Data are means ± standard deviation of three biological samples.

To study the biological role of *JrGA20ox1* in drought stress responses, transformed walnut plants that overexpressed *JrGA20ox1* (OE) and plants with RNA interference of *JrGA20ox1* (RNAi) were constructed. The embryonic shoots were excised after 4 weeks of culture and micropropagated every 2 weeks. Three OE lines and three RNAi lines were selected for further study (Fig. 1A). qRT–PCR results showed that the relative expression of *JrGA20ox1* was highly significantly upregulated in the OE lines but significantly downregulated in all RNAi lines compared with the WT plants (Fig. 1B).

Further analysis showed that OE and RNAi regenerated plants exhibited growth alteration compared with WT after 2 weeks of culture (Fig. 1A). The average height of OE lines was significantly higher than that of WT (Fig. 1C). For RNAi, plant height was significantly lower compared with WT (Fig. 1C). However, the internode number among all studied lines showed no significant difference (Fig. 1D). The OE lines had the highest average internode length followed by WT and RNAi lines (Supplementary Data Fig. S1). Therefore, the RNAi lines exhibited a semi-dwarf phenotype with shorter internodes. In addition, there was no significant difference in either fresh weight or dry weight among WT, RNAi, and OE lines (Fig. 1E and F).

### *JrGA20ox1* negatively regulates drought tolerance of walnut

**Figure 2:**
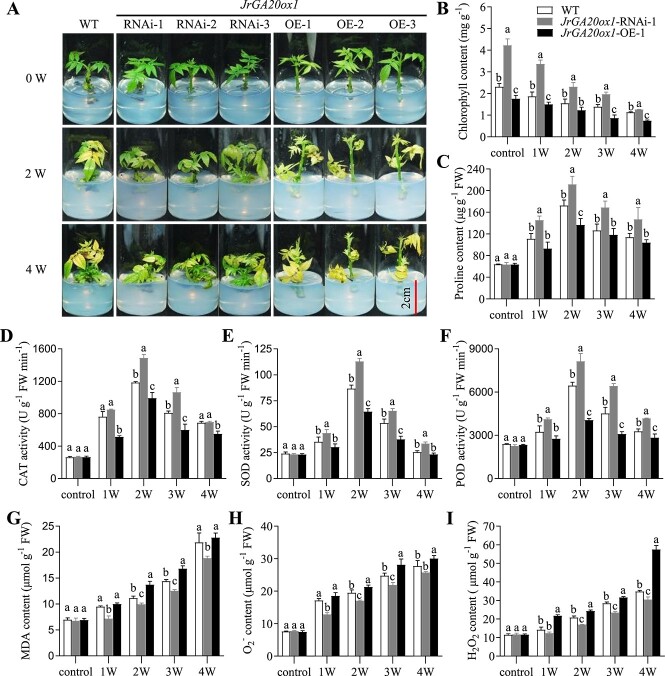
*JrGA20ox1* negatively regulates walnut drought tolerance. **A** Phenotypes of WT, *JrGA20ox1*-RNAi, and *JrGA20ox1*-OE plants under 5% PEG stress. **B**, **C** Contents of chlorophyll (**B**) and proline (**C**) in WT, *JrGA20ox1*-RNAi-1, and *JrGA20ox1*-OE-1 plants under 5% PEG stress for 0 (control), 1, 2, 3, and 4 weeks. **D**–**F** Activities of CAT (**D**), SOD (**E**), and POD (**F**) in WT, *JrGA20ox1*-RNAi-1, and *JrGA20ox1*-OE-1 plants under 5% PEG stress for 0 (control), 1, 2, 3, and 4 weeks. **G**–**I** Contents of MDA (**G**), O_2_^−^ (**H**), and H_2_O_2_ (**I**) in WT, *JrGA20ox1*-RNAi-1, and *JrGA20ox1*-OE-1 plants under 5% PEG stress for 0 (control), 1, 2, 3, and 4 weeks. Different letters indicate significant differences (*P* < 0.05) using one-way ANOVA with Tukey’s test for multiple comparisons. Data are means ± standard deviation of three biological samples.

For further assay of drought tolerance, the OE, RNAi, and WT lines were grown in DKW medium supplemented with (simulating drought stress) or without 5% PEG for 4 weeks. The OE leaves showed widespread yellowing and began to fall while RNAi leaves remained green after PEG treatment for 2 weeks. The OE and WT leaves turned yellow and fell in large numbers, whereas the RNAi leaves were just beginning to turn yellow after 4 weeks of culture (Fig. 2A). The leaves of OE, RNAi, and WT plants lines remained green under normal conditions. We selected RNAi-1 and OE-1 lines for further analysis. The chlorophyll content was affected by *JrGA20ox1* expression and its change was consistent with the phenotypic changes (Fig. 2B, Supplementary Data Fig. S2A).

To study whether the altered drought tolerance mediated by *JrGA20ox1* is due to a change in osmotic potential, the proline contents were also determined. Proline levels increased in the first 2 weeks of culture and followed by a decrease under PEG stress in all lines (Fig. 2C). The RNAi-1 plants had the highest proline levels followed by WT and OE-1 at 1–4 weeks of culture (Fig. 2C). There was no significant difference among the OE, RNAi, and WT lines under normal conditions (Supplementary Data Fig. S2B).

The activities of superoxide dismutase (SOD), peroxidase (POD), and catalase (CAT) were also measured. All three enzyme activities increased in the first 2 weeks of culture, followed by a gradual decrease under PEG stress in all plant lines (Fig. 2D–F). Further analysis showed that RNAi-1 had the maximum activity levels of these enzymes at every culture stage, followed by WT and OE-1 (Fig. 2D–F). There were no significant differences among all lines under normal growth conditions (Supplementary Data Fig. S2C–E).

The level of malondialdehyde (MDA) is commonly known as a marker of oxidative stress and antioxidant status in the cells. The results showed that there were no significant differences in MDA accumulation among all plant lines under normal growth conditions (Supplementary Data Fig. S2F). However, with the extension of PEG stress, MDA levels increased gradually in all the studied lines. The OE-1 plants accumulated the highest levels of MDA followed by WT and RNAi-1 at 1–4 weeks of culture, although there was no significant difference between OE-1 and WT at 1 and 4 weeks (Fig. 2G). H_2_O_2_ and O_2_^−^ were also examined in WT, RNAi-1, and OE-1 lines and the trend was similar to that of MDA (Fig. 2H and I, Supplementary Data Fig. S2G and H).

In addition, the natural drought stress assay was performed to further validate the effect of *JrGA20ox1* on walnut drought tolerance. Results showed that the phenotype, the contents of chlorophyll, proline, MDA, H_2_O_2_, and O_2_^−^, and the activities of CAT, SOD, and POD in WT, RNAi-1, and OE-1 lines showed trends similar to those of the 5% PEG-­treated plants (Supplementary Data Fig. S3A–I). These results suggested that *JrGA20ox1* negatively regulates drought tolerance of walnut.

### *JrGA20ox1*-transformed rootstocks negatively regulate the drought response in grafted wild-type scions in walnut

**Figure 3:**
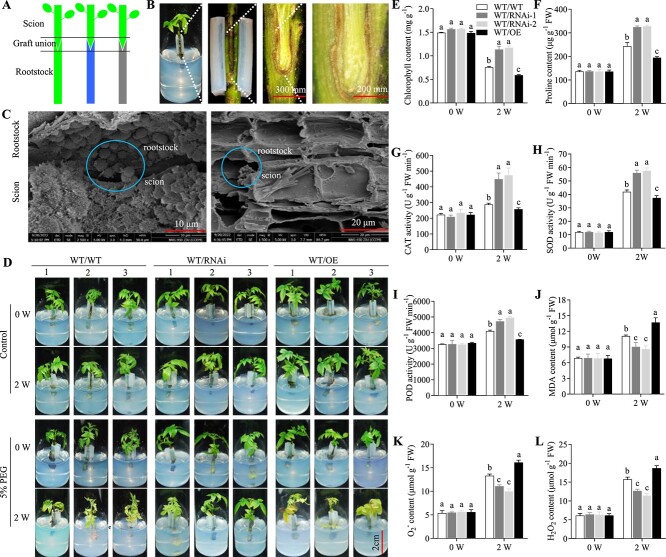
Effect of *JrGA20ox1*-transformed rootstocks on WT walnut scions. **A** Schematic diagram of walnut in vitro grafting combinations. Green indicates WT, blue indicates *JrGA20ox1*-RNAi line, and gray indicates *JrGA20ox1*-OE line. **B** Well-healed graft union. **C** SEM image of a graft junction. (Left) Callus tissue proliferates from both stems; the blue circle indicates callus cells from both stems are sealing the gap between stock and scion and re-establishing intercellular communication. (Right) Part of graft junction is well healed; the blue circle indicates the part of graft junction that is healing through callus tissue proliferation. **D** Phenotypes of WT/WT, WT/RNAi, and WT/OE lines under 0% (control) and 5% PEG stress. **E**, **F** Contents of chlorophyll (**E**) and proline (**F**) in scions of WT/WT, WT/RNAi, and WT/OE lines under 5% PEG stress for 0 and 2 weeks. **G**–**I** Activities of CAT (**G**), SOD (**H**), and POD (**I**) in scions of WT/WT, WT/RNAi, and WT/OE lines under 5% PEG stress for 0 and 2 weeks. **J**–**L** Contents of MDA (**J**), O_2_^−^ (**K**), and H_2_O_2_ (**L**) in scions of WT/WT, WT/RNAi, and WT/OE lines under 5% PEG stress for 0 and 2 weeks. Different letters indicate significant differences (*P* < 0.05) using one-way ANOVA with Tukey’s test for multiple comparisons. Data are means ± standard deviation of three biological samples.

Cleft *in vitro* grafting was used to investigate the effect of *JrGA20ox1* gene on survival rate (Fig. 3A). Callus began developing at the graft junction 3–4 days after *in vitro* grafting. The cleft *in vitro* grafting survival percentage was 85–95% in all groups. The result showed there were no significant differences in all *in vitro* grafting combinations. For further histological observation, the graft junction was cut vertically through the pith. The scion and rootstock of the graft union healed smoothly, indicating that *JrGA20ox1* expression had no negative effect on walnut *in vitro* grafting (Fig. 3B). Further investigation into cellular events during graft union formation was conducted using scanning electron microscopy (SEM). Within a few days, callus tissue formed at the graft site, closing the wound and establishing a physical connection between the graft partners (Fig. 3C).

In order to investigate the effect of *JrGA20ox1*-transformed rootstocks on the drought response in grafted WT scions, WT/OE (scion/rootstock), WT/RNAi and WT/WT grafted plants were grown in DKW medium supplemented with (simulating drought stress) or without PEG for 2 weeks (Fig. 3D). The scion leaves of WT/OE, WT/RNAi, and WT/WT grafted plants remained green under normal conditions. When exposed to PEG conditions for 2 weeks, the scion leaves of WT/OE showed widespread yellowing and began to fall and the leaves of WT/WT scion began to turn yellow, while RNAi scion leaves of WT/RNAi remained fresh (Fig. 3D). The chlorophyll content in all grafted groups decreased gradually with the extension of PEG stress (Fig. 3E). The WT/RNAi lines exhibited significantly higher chlorophyll levels and WT/OE lines showed a significantly lower level compared with WT/WT at 2 weeks of culture (Fig. 3E). Proline content increased gradually with the extension of PEG stress in all grafted groups. The maximum content of proline was observed in WT/RNAi followed by WT/WT and WT/OE at 2 weeks of culture (Fig. 3F). The enzyme activities of CAT, SOD, and POD and the contents of MDA, O_2_^−^, and H_2_O_2_ showed changes similar to those of proline content (Fig. 3G–L). Under normal conditions, the chlorophyll content in all grafted groups decreased slightly while the SOD activity and contents of O_2_^−^ and H_2_O_2_ in all grafted groups increased within 2 weeks of culture (Supplementary Data Fig. S4). We observed that almost all physiological indexes had no significant differences among WT, RNAi, and OE lines at 0 or 2 weeks of culture under normal conditions except for the chlorophyll content in WT/OE at 2 weeks of culture (Supplementary Data Fig. S4A–H).

To further validate the effect of *JrGA20ox1*-transformed rootstocks on non-transgenic walnut scions under drought stress, the natural drought stress assay was also performed. Results showed that the phenotype, the contents of chlorophyll, proline, MDA, H_2_O_2_, and O_2_^−^, and the activities of CAT, SOD, and POD in WT/WT, WT/RNAi, and WT/OE lines showed trends similar to those of 5% PEG-treated grafted plants (Supplementary Data Fig. S5A–I). These results suggested that *JrGA20ox1*-transformed rootstocks negatively regulate the drought response in grafted WT scions in walnut.

### Gibberellins but not *JrGA20ox1* move from *JrGA20ox1*-OE rootstock to wild-type scion

**Figure 4:**
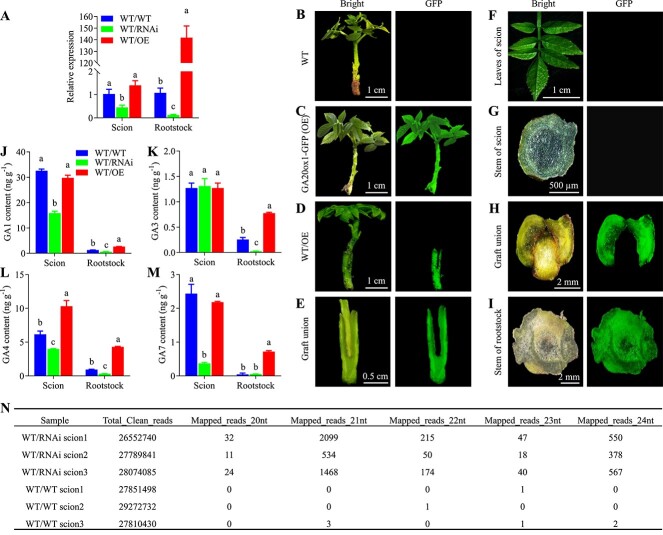
Movement of signals from *JrGA20ox1*-OE and *JrGA20ox1*-RNAi rootstocks to WT scions. **A** qRT–PCR analysis of *JrGA20ox1* expression in scions of WT/WT, WT/RNAi, and WT/OE lines under 5% PEG stress for 2 weeks. **B**–**I** Confocal images of WT (**B**), *GA20ox1*-OE (**C**), WT/OE (**D**), longitudinal section of graft junction from WT/OE (**E**), leaves from scion of WT/OE (**F**), cross-section of scion stem from WT/OE (**G**), cross-section of graft union from WT/OE (**H**), and cross-section of rootstock stem from WT/OE (**I**). GFP fluorescence and bright-field images were captured by confocal laser scanning. **J**–**M** Concentrations of GA_1_ (**J**), GA_3_ (**K**), GA_4_ (**L**), and GA_7_ (**M**) in scions and rootstocks after 5% PEG treatment for 2 weeks. Different letters indicate significant differences (*P* < 0.05) using one-way ANOVA with Tukey’s test for multiple comparisons. Data are means ± standard deviation of three biological samples. **N** Fractions of 20–24-nt sRNAs mapped to *JrGA20ox1*-hpRNA region in scions of WT/RNAi and WT/WT after 5% PEG treatment for 2 weeks.

To investigate whether the changes in drought tolerance of grafted WT scion were caused by the movement of *JrGA20ox1* from the rootstock, we first examined the expression levels of *JrGA20ox1*. Results showed that there were no significant changes in *JrGA20ox1* transcript level between the scions of WT/WT and WT/OE, while there was a significant decrease in scions of WT/RNAi compared with WT/WT (Fig. 4A). To investigate the possibility of the movement of *JrGA20ox1* between rootstock and grafted WT scion, we tested the GFP signals of rootstock and scion. The results showed that strong GFP signals were present in the *JrGA20ox1*-OE-transformed rootstock, but no signals were detected in the scion (Fig. 4B–I). These results suggested that both the mRNA and protein of *JrGA20ox1* could not move from the rootstock to the grafted WT scion.

Previous studies have revealed that GA can move over long distances [32, 33], even between rootstock and scion, in grafted *Arabidopsis* [34]. This observation prompted us to investigate whether the translocation of GA between rootstock and scion contributes to drought tolerance of the grafted walnut. To investigate this possibility, we measured the active GA content in the rootstocks and scions after PEG-simulated drought stress treatment. Strikingly, only the GA_4_ content was significantly increased in the scions of WT/OE, while GA_1_, GA_4_, and GA_7_ were significantly decreased in the scions of WT/RNAi compared with WT/WT (Fig. 4J–M). All the GAs were significantly increased in rootstocks of WT/OE, while all the GAs were significantly decreased in the rootstocks of WT/RNAi compared with WT/WT (Fig. 4J–M). These results suggested that GA_4_ may move from *JrGA20ox1*-OE rootstock to WT scion under drought stress.

### Rootstock-to-scion movement of sRNA contributes to decreased gibberellin content in WT/RNAi scions

The decreased GA content in WT/RNAi scions led us to speculate that sRNAs produced by the RNAi vector expressing short hpRNAs of the genomic *JrGA20ox1* were capable of moving from *JrGA20ox1*-RNAi transformed rootstocks to WT scions, which further led to a successive decline in *JrGA20ox1* expression and GA content. To test this hypothesis, we sequenced the sRNA of scions from WT/RNAi and WT/WT under drought stress. To compare the profiles of hpRNA-derived siRNAs, the sequencing reads from the scions of WT/WT and WT/RNAi were quality-filtered and mapped to a reference fragment of the *JrGA20ox1* gene sequence, from which the hpRNA was derived. The results showed that hpRNA-specific sRNAs in the scions of WT/RNAi exhibited much higher abundance than those in the scions of WT/WT (Fig. 4N, Supplementary Data Table S1), suggesting that hpRNA-derived sRNAs in transformed rootstocks were transported to the WT walnut scions. In the scions of WT/RNAi, the most prevalent hpRNA-specific sRNAs were 21-nt sRNAs (66%) followed by 24-nt ones (24%) (Fig. 4N, Supplementary Data Table S1). The GA_1_, GA_4_, and GA_7_ contents were significantly decreased in the scions of WT/RNAi compared with WT/WT (Fig. 4J, L and M) and the *JrGA20ox1* transcript level was significantly lower in the scions of WT/RNAi compared with WT/WT (Fig. 4A). We concluded that rootstock-to-scion movement of sRNA contributes to the decreased GA content in the scions of WT/RNAi.

### Transformed rootstock positive regulates multiple GA-responsive genes in grafted wild-type scions in walnut

**Figure 5:**
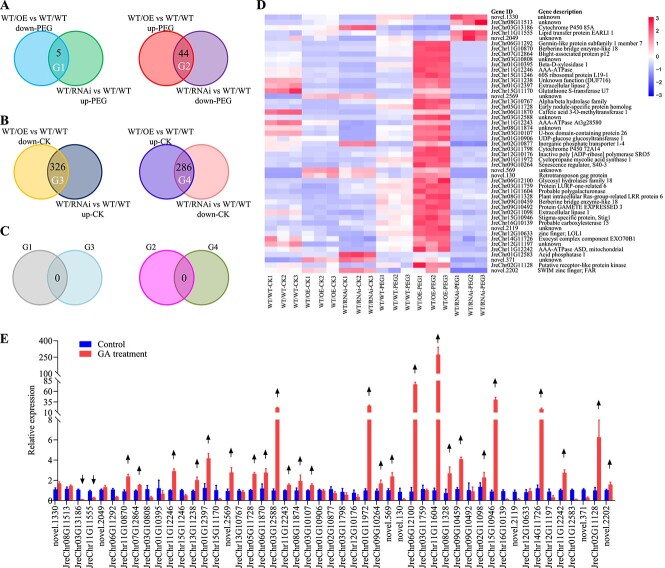
Transformed rootstock positively regulates multiple GA-responsive genes in grafted WT scions in walnut. **A** Overlap of differentially expressed genes (DEGs) in scions from WT/OE and WT/RNAi compared with WT/WT under 5% PEG stress for 2 weeks. The left panel indicates the overlap of downregulated genes of WT/OE vs WT/WT and upregulated genes of WT/RNAi vs WT/WT (G1); the right panel indicates the overlap of the upregulated genes of WT/OE vs WT/WT and the downregulated genes of WT/RNAi vs WT/WT (G2). **B** Overlap of DEGs in scions from WT/OE and WT/RNAi compared with WT/WT under normal growth condition for 2 weeks. The left panel indicates the overlap of the downregulated genes of WT/OE vs WT/WT and the upregulated genes of WT/RNAi vs WT/WT (G3); the right panel indicates the overlap of the upregulated genes of WT/OE vs WT/WT and the downregulated genes of WT/RNAi vs WT/WT (G4). **C** Overlap of G1 and G3 (left panel) and overlap of G2 and G4 (right panel). **D** Relative expression and annotation of the 49 genes from G1 and G2. **E** qRT–PCR analysis of the response of the 49 genes to GA. Downward and upward arrows indicate genes downregulated and upregulated by GA, respectively.

To discover key genes contributing to the enhanced drought tolerance phenotype, we conducted RNA sequencing in grafted WT scions of WT/RNAi, WT/WT, and WT/OE. Compared with the PEG-treated scions of WT/WT, 2792 upregulated and 796 downregulated genes were identified in the PEG-treated scions of WT/OE (Supplementary Data Tables S2 and S3), whereas 3003 upregulated and 1070 downregulated genes were identified in the PEG-treated scions of WT/RNAi (Supplementary Data Tables S4 and S5). In addition, 5 genes were downregulated in the PEG treated scions of WT/OE but upregulated in the PEG-treated scions of WT/RNAi (G1), and 44 genes were upregulated in the PEG-treated scions of WT/OE but downregulated in the PEG-treated scions of WT/RNAi (G2) (Fig. 5A). In control conditions, 326 genes were downregulated in the PEG-treated scions of WT/OE but upregulated in the PEG-treated scions of WT/RNAi (G3), and 286 genes were upregulated in the PEG-treated scions of WT/OE but downregulated in the PEG-treated scions of WT/RNAi (G4) (Fig. 5B, Supplementary Data Tables S6 and S7). However, there are no overlaps between G1 and G3 as well as between G2 and G4 (Fig. 5C), suggesting that the 49 genes were regulated in an opposite manner in the scions of WT/OE and WT/RNAi under PEG-simulated drought stress but not under normal condition (Fig. 5D).

The change of GA content in scions prompted us to further test whether the expression of these 49 genes was also induced by GA. The qRT–PCR data showed that the number of genes regulated by GA was 27 out of 49 (Fig. 5E). This result suggested that other signals, except for GAs, may be moved from rootstock to scion to regulate the GA non-responsive genes in the scion under PEG-simulated drought stress. It is worth noting that there are two GA-upregulated genes, *S40-3* and *Exo70B1*, and one GA-non-responsive gene, *LOL1*, that may partly contribute to the drought resistance of the scion (Fig. 5D), because their homologous genes in other plants have been proved to be involved in senescence, stomatal opening, or programmed cell death [35–37]. We further verified that LOL1 and FAR1 could regulate the expression of multiple genes from the 49 genes by the dual-luciferase assay, which provided some signal clues of drought resistance in walnut (Supplementary Data Fig. S6).

### *JrGA20ox1*-transformed rootstocks regulate multiple drought-related indexes of wild-type scions

**Figure 6:**
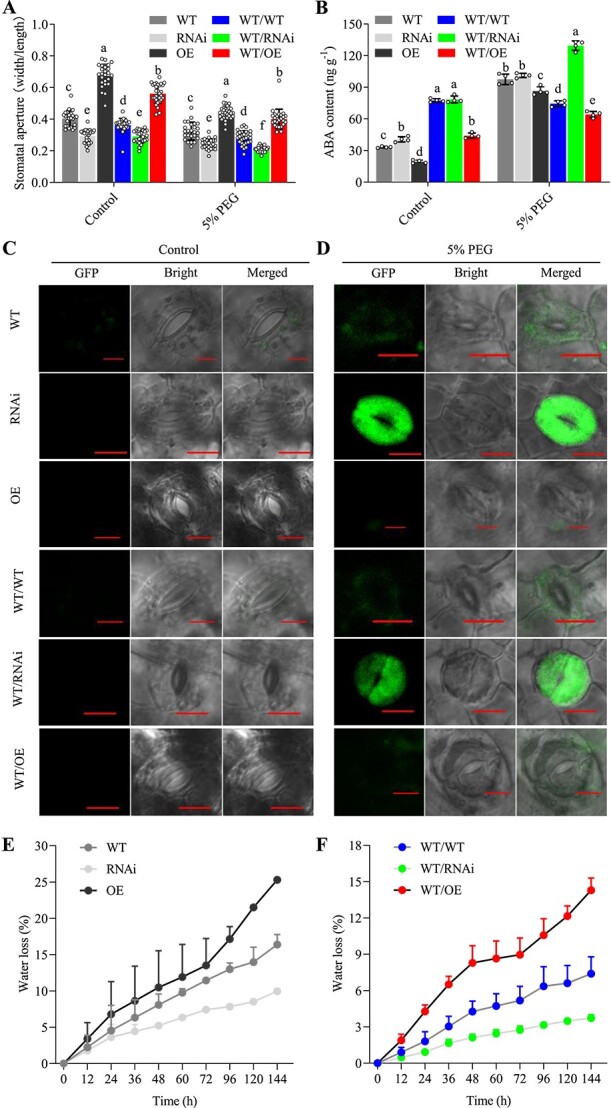
*JrGA20ox1*-transformed rootstocks involved in drought response in WT scions. **A** Statistics of stomatal aperture of WT, *JrGA20ox1*-RNAi (RNAi), and *JrGA20ox1*-OE (OE) lines under 0% (control) and 5% PEG treatments for 1 week. Different letters indicate significant differences (*P* < 0.05) using one-way ANOVA with Tukey’s test for multiple comparisons. **B** Statistics of stomatal aperture of scion leaves from WT/WT, WT/RNAi, and WT/OE lines under 0% (control) and 5% PEG treatments for 1 week. Different letters indicate significant differences (*P* < 0.05) using one-way ANOVA with Tukey’s test for multiple comparisons. **C** Comparison of ROS levels in guard cells under 0% (control) PEG treatment detected with H_2_DCF-DA. **D** Comparison of ROS levels in guard cells under 5% PEG treatment detected with H_2_DCF-DA. **E** Water loss rates for WT, RNAi, and OE lines during 1 week of treatment with 5% PEG. **F** Water loss rates for WT/WT, WT/RNAi, and WT/OE lines during 1 week of treatment with 5% PEG. Data are means ± standard deviation of three biological samples.

We have proved that *JrGA20ox1*-OE rootstocks positively while *JrGA20ox1*-RNAi rootstocks negatively regulate the senescence and programmed cell death of WT scions by comparing their chlorophyll content, ROS accumulation, and antioxidant enzyme activities (Fig. 3E–L). We next asked whether *JrGA20ox1*-transformed rootstocks affect the stomatal aperture of WT scions dependent on or independently of ABA. As expected, scions from WT/OE displayed significantly larger stomatal apertures and lower ABA content while scions from WT/RNAi displayed significantly smaller stomatal apertures and higher ABA content than scions from WT/WT under both control and PEG-simulated drought stress conditions, which is consistent with the non-grafted *JrGA20ox1*-transformed plants (Fig. 6A and B, Supplementary Data Fig. S7A and B). A previous study has reported that ROS production in guard cells could promote stomatal closure and result in enhanced drought resistance [38]. We also surprisingly found that the ROS in guard cells from 5% PEG-treated RNAi and WT/RNAi plants were much higher than in those from RNAi and WT/RNAi plants under normal growth condition, but ROS fluorescence in guard cells from other lines was hardly observed under both normal growth condition and PEG stress (Fig. 6C and D). These results suggested that *JrGA20ox1*-RNAi and WT/RNAi plants may partly participate in drought resistance by reducing stomatal opening and thus reducing water loss and transpiration rate. Our water loss and transpiration rate analyses indeed revealed that *JrGA20ox1*-OE and WT/OE plants lose more water and have higher transpiration rates, while *JrGA20ox1*-RNAi and WT/RNAi plants lose less water and have lower transpiration rates than control plants under PEG-simulated drought stress, which is also consistent with the non-grafted *JrGA20ox1*-transformed plants (Fig. 6E and F, Supplementary Data Fig. S8).

## Discussion

Grafting has been employed to investigate long-distance signaling in various processes, such as shoot branching [39] and stress responses [40]. The current study demonstrates long-distance drought regulation signaling by integrating the transformed technology and micrografting of walnut seedlings in test tubes for the first time in trees, which will provide a reference for the accurate and in-depth study of tree grafting. Our results have revealed the influence of rootstocks transformed with a GA synthesis pathway gene, *JrGA20ox1*, on the drought resistance of WT scions. A plausible idea is that the movement of *JrGA20ox1* mRNAs between rootstock and scion could be responsible for changing the drought resistance in WT scions. This is because a growing body of work has demonstrated the long-distance movement of RNA. For example, studies have shown mRNA movement across graft junctions in pumpkin/cucumber heterografts [41], Chinese pear/wild pear heterografts [42], and grafts of different *Arabidopsis* ecotypes [43]. However, our results showed that there is no difference in *JrGA20ox1* mRNA abundance among WT scions from WT/WT, WT/OE, and WT/RNAi, and no GFP signals were visible in WT scions when WT scions were grafted onto *JrGA20ox1*-GFP-overexpressing rootstocks, suggesting that there is no movement of *JrGA20ox1* and its protein between rootstock and scion (Fig. 4). A recent study using an experimental method similar to ours found the opposite results in *Arabidopsis*, i.e. when GFP-CEPDL2 scions were grafted onto WT rootstocks, accumulation of GFP-CEPDL2 signals in roots was detected [44]. These results suggest that the movement of mRNAs or proteins between scion and rootstock is selective.

It is worth noting that previous studies have revealed that GA_12_ can move from rootstock to scion, which is then converted to GA_4_ by the activities of GA 20-oxidases and GA 3-oxidases, and then the GA_4_ contributes substantially to temperature-induced shoot growth [34, 45]. Similarly, our study shows that GA_4_ accumulated to higher levels in WT scions of WT/OE grafts compared with WT/WT grafts under PEG-simulated drought stress (Fig. 4). However, we are not sure whether this increased GA_4_ has moved from the rootstock or is derived by conversion of GA_12_ translocated from the rootstock to the scion, because the experimental system using walnut is not as easy to establish as systems using model plants such as *Arabidopsis thaliana*, and this requires further study in the future.

Micrografts of WT scion into *JrGA20ox1*-RNAi-transformed rootstocks enable an enhancement of drought tolerance of WT scions that is also observed in *JrGA20ox1*-RNAi-transformed non-grafted walnut. These results suggest that the signals responsible for enhancement of drought tolerance moved from *JrGA20ox1*-RNAi-transformed rootstock to the WT scion. To uncover which signals that travel through graft junctions enhance the drought tolerance of WT scion, we first measured the GA content. As we expected, the bioactive GAs, such as GA_1_ and GA_4_, accumulated to lower levels in WT scions of WT/RNAi grafts compared with WT/WT grafts under PEG-simulated drought stress, and this finding could therefore partly explain the difference in drought tolerance. It is easy to assume that sRNAs produced by the RNAi vector expressing short hpRNAs of the genomic *JrGA20ox1* were capable of moving from *JrGA20ox1*-RNAi-transformed rootstocks to WT scions, which further led to a successive decline in *JrGA20ox1* expression and GA content. This is because a growing body of studies have proved that small RNAs could move from scion to rootstock in grafted plants [46, 47]. Especially, the transfer of transgene-derived siRNAs from transformed cherry rootstocks to non-transformed scions in grafted trees has been demonstrated [12]. In line with these discoveries, our findings also indicated that the sRNAs produced by an RNAi vector expressing short hpRNAs of genomic *JrGA20ox1* were capable of moving from *JrGA20ox1*-RNAi-transformed rootstocks to WT scions. The reduced *JrGA20ox1* mRNA abundance in scions of WT/RNAi further confirms our hypothesis.

We have demonstrated that WT/RNAi, resistant to drought, exhibited lower GA content in its WT scions, whereas WT/OE, sensitive to drought, displayed higher GA content in its WT scions, compared with WT/WT. However, how do the changes in GA affect the drought phenotype? Previous studies have demonstrated that decreased GA levels lead to the activation of various stress-related genes [48] and accumulation of osmolytes [49] and ROS-scavenging enzymes [50], all related to drought tolerance. In tomato, suppressing GA accumulation reduced water loss under water-deficit conditions [51]. Drought reduced GA accumulation by downregulating *GA20ox1* and *GA20ox2* etc., resulting in inhibited leaf growth and stimulating ABA-induced stomatal closure during early soil dehydration stages [26]. Similarly, our study showed that WT/RNAi increased while WT/OE decreased the activity of ROS-scavenging enzymes, ABA content, and stomatal closure in WT scions compared with WT/WT under PEG-simulated drought stress. ROS production in guard cells is involved in drought resistance through promoting stomatal closure [38]. ABA regulates stomatal movement under drought stress depending on ROS [52]. However, as far as we know, there are no reports on the relationship between GA and ROS production in guard cells. Here, we found that ROS production in guard cells is highly associated with active GA content but not ABA content under PEG-simulated drought stress (Figs 4J–M and 6B–D). These results indicate that GA may regulate stomatal movement not only through the ABA pathway but also through other pathways.

We identified 49 genes regulated in an opposite manner in the PEG-treated scions of WT/OE and WT/RNAi, of which 27 genes respond to GA treatment. These results indicate that drought resistance of the scion is regulated not only by GA signal, but also by other signals, which may be moved from the rootstock. Strikingly, there are two GA-upregulated genes, *S40-3* and *Exo70B1*, and one GA non-responsive gene, *LOL1*, that may partly contribute to the drought resistance of the scion. This is supported by the following reported clues. First, the *AtS40-3* gene showed a much higher expression level in senescent leaves compared with non-senescent ones in *Arabidopsis* WT plants, and delayed senescence was observed in the *AtS40-3a* mutant compared with the WT [35]. Second, it was found that *Exo70B1* negatively regulates stomatal opening in *Arabidopsis* [36]. Third, LOL1 has been identified as a positive regulator of programmed cell death in *Arabidopsis* through the regulation of ROS homeostasis and Cu/Zn SOD activity [37].

**Figure 7:**
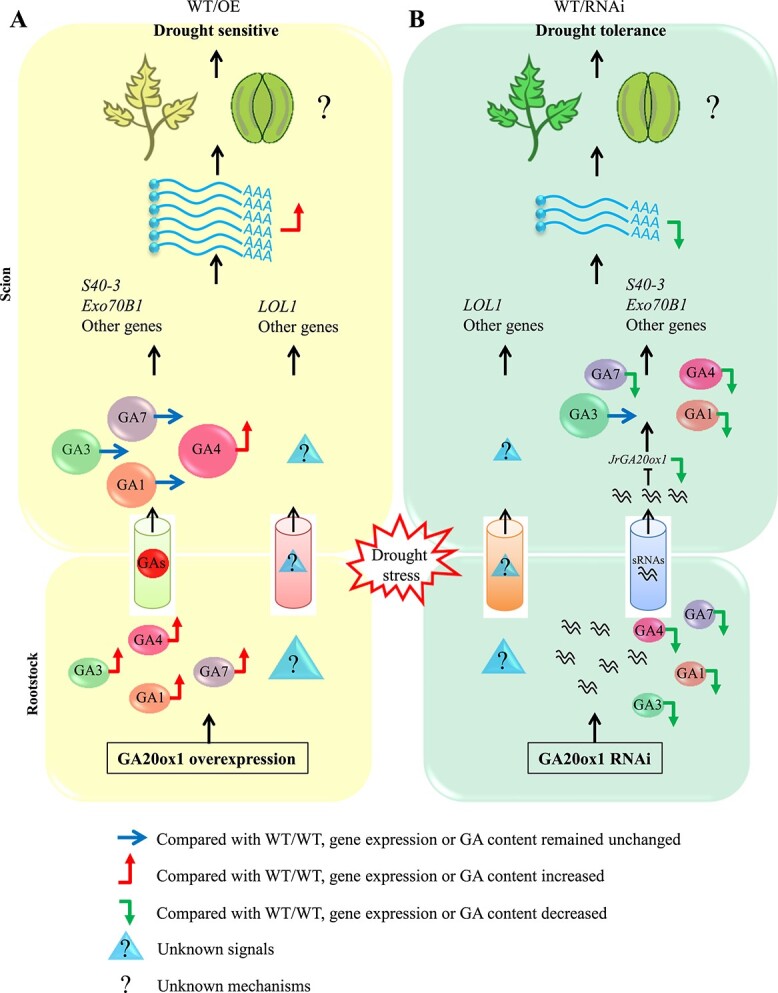
Model of *JrGA20ox1*-transformed rootstock regulation of drought stress in WT scions. **A** When WT/OE plants suffer drought stress, synthesis of active GAs increases in the rootstock. The subsequent GA_4_ and unknown signals move from the rootstock to the WT scion, and can induce the expression of a series of genes including *S40-3*, *Exo70B1*, and *LOL1*. Then, ROS are excessively accumulated in leaves, leaf stomata open wider, and more water is lost in the scion of WT/OE than in WT/WT; thereby, WT/OE is more drought-sensitive than WT/WT. **B** When WT/RNAi plants suffer drought stress, the synthesis of active GAs decreases in the rootstock. The sRNAs produced by the RNAi vector expressing short hpRNAs of genomic *JrGA20ox1* move from *JrGA20ox1*-RNAi-transformed rootstocks to WT scions, which further leads to a successive decline in *JrGA20ox1* expression and active GA content. Meanwhile, unknown signals move from the rootstock to the WT scion. The decreased active GAs and unknown signals further lead to the lower expression of a series of genes, including *S40-3*, *Exo70B1*, and *LOL1*. Then, ROS are less accumulated in leaves and massively induced in guard cells, leaf stomata are more closed, and less water is lost in the scion of WT/RNAi than in WT/WT; thereby, WT/RNAi is more drought-tolerant than WT/WT.

Based on this work, and the work of others, we propose the following model for *JrGA20ox1*-transformed rootstock regulation of drought stress in WT scions (Fig. 7). When WT/OE plants suffer drought stress, the synthesis of active GAs increases in the rootstock. The subsequent GA_4_ and unknown signals move from the rootstock to the WT scion, and they can induce the expression of a series of genes, including *S40-3*, *Exo70B1*, and *LOL1*. Then, ROS in leaves are excessively accumulated, leaf stomata open wider, and more water is lost in the scion of WT/OE than in WT/WT plants; thereby, WT/OE is more drought-sensitive than WT/WT (Fig. 7A). When WT/RNAi plants suffer drought stress, the synthesis of active GAs decreases in the rootstock. The sRNAs produced by the RNAi vector expressing short hpRNAs of genomic *JrGA20ox1* move from *JrGA20ox1*-RNAi transformed rootstocks to WT scions, which further leads to a successive decline in *JrGA20ox1* expression and active GA content. Meanwhile, unknown signals move from the rootstock to the WT scion. The decrease in active GAs and unknown signals further lead to the lower expression of a series of genes, including *S40-3*, *Exo70B1*, and *LOL1*. Then, ROS are less accumulated in leaves and massively induced in guard cells, leaf stomata are more closed, and less water is lost in the scion of WT/RNAi than in WT/WT plants; thereby, WT/RNAi is more drought-tolerant than WT/WT (Fig. 7B).

## Materials and methods

### Vector construction

To construct the *JrGA20ox1*-GFP plasmid, the coding sequence of *JrGA20ox1* without stop codon was connected to the pCAMBIA1300-GFP vector. The *JrGA20ox1*-RNAi plasmid was created by inserting a 200-bp cDNA fragment of *JrGA20ox1* into the pTCK303 vector between SacI and SpeI (as antisense fragment). Thereafter, the 200-bp cDNA fragment of *JrGA20ox1* was ligated into the pTCK303 vector between KpnI and BamHI. Primers are listed in Supplementary Data Table S8.

### Generation of transformed walnut lines and plant growth conditions

Somatic embryos were derived from walnut hybrid line *Juglans hindsii* × *J. regia*. Hybrid seedlings are known as ‘Zhongningsheng’ and are characterized as promising walnut rootstock in China [53]. *JrGA20ox1*-GFP and *JrGA20ox1*-RNAi plasmids were introduced into walnut somatic embryos separately using an *Agrobacterium tumefaciens*-mediated transformation method according to our previous study [54]. Transformed somatic embryos were transferred into shoot multiplication medium for germination. The generated shoots were then incised and multiplied by subculturing at 2-week intervals. Walnut somatic embryos and regenerated shoots were tissue-cultured according to a previous protocol [54]. Cultures was grown at 25 ± 2°C, in darkness for somatic embryos and with a 16-h photoperiod supplied by white fluorescent light (3000 lx) for shoot growth.

### qRT–PCR analysis

Total RNA was extracted using a Plant Total RNA Kit (DP432, Tiangen, China). cDNA synthesis utilized PrimeScript RT Master Mix (Takara, Japan). The qRT–PCR reaction utilized a C1000 Touch™ Thermal Cycler system from Bio-Rad (USA) and ChamQ SYBR qPCR Master Mix from Vazyme (China), along with gene-specific primers (Supplementary Data Table S8). The qRT–PCR conditions were as follows: 95°C for 2 min followed by 45 cycles of 95°C for 10 s, 57°C for 10 s, and 72°C for 20 s. Relative expression levels were determined and normalized against *JrActin2* expression by the 2^−ΔΔCT^ method.

### Protocol for *in vitro* grafting

*In vitro* grafting protocol were divided into three groups: (i) WT/WT (control), (i) WT/OE, and (iii) WT/RNAi. Two-week old tissue-cultured shoots were utilized for *in vitro* cleft grafting according to a previous protocol [15]. WT scions (2 cm from tip to base) were *in vitro* grafted on *JrGA20ox1*-OE, *JrGA20ox1*-RNAi, and WT rootstocks (2 cm from base to tip). The graft union was enclosed using a sterilized grafting clip. Grafted plants were tissue-cultured on solidified DKW basal medium with the addition of BAP and IBA, exposed to cool white fluorescent light with a 16:8 day-length cycle for a duration of 2 weeks. Grafts with etiolated or dead scions were discarded and well healed grafts were used for further study.

### PEG-simulated drought stress and the growth response assays

To determine the effects of drought stress on *in vitro* shoots, 5% PEG8000 was added to shoot multiplication medium (solidified DKW basal medium containing BAP and IBA). Plants with 2-cm non-grafted shoots or 2-week-grafted plants were cultured in a growth chamber at 25 ± 2°C with daily cycle of 16 h light and 8 h darkness. Natural drought stress was also included. Cultures with ~6-cm non-grafted shoots or 2-week-grafted plants were taken out of the test tube and cultures without culture medium were placed on gauze in the above growth chamber for natural drought stress. Fresh weight (FW), dry weight (DW), shoot length, node number, and chlorophyll content were evaluated to assess the growth response.

### Stomatal aperture and water loss and transpiration rate assays

To measure stomatal aperture, the epidermis was stripped from the surface of the leaves. Stomatal aperture was captured using a light microscope (Olympus BX60). Measurements were performed using the free software Image-Pro Plus 6.0. To assess water loss rate under dehydration conditions, both non-grafted and grafted plants were subjected to air exposure at room temperature and weighed at specified intervals. Transpiration rate was measured by directly exposing the plants to ambient air and recording their weight changes every minute using a micro-analytical balance. The transpiration rate was calculated based on the water loss during each interval and converted to a per hour basis.

### Antioxidant enzyme activity assay

Antioxidant enzyme activities were measured using an assay kit (Suzhou Keming Biotech Co. Ltd, Jiangsu, China) following the provided instructions. For SOD assessment, a reaction mixture was prepared containing 50 mM Na_3_PO_4_ buffer, 0.1 mM EDTA-Na2, 130 mM methionine, 0.75 mM NBT, and 0.1 mM riboflavin. Subsequently, 50 μg of enzyme extract was added and incubated for 20 min, followed by recording the absorbance at 560 nm. For guaiacol POD activity determination, a reaction mixture comprising 0.2 ml of enzyme extract, 2.5 ml of 0.025 mol l^−1^ guaiacol, and 0.2 ml of 0.25 mol l^−1^ H_2_O_2_ was incubated. The increase in absorbance at 470 nm was then measured at 25°C. For CAT activity determination, a mixture comprising 50 mM Na_3_PO_4_ buffer, 15 mM H_2_O_2_, and 5 μg of enzyme extract was used, and the decomposition of H_2_O_2_ was measured at 240 nm.

### Determination of hydrogen peroxide and superoxide anion concentrations

Concentrations of H_2_O_2_ and O_2_^−^ were measured utilizing assay kits (Suzhou Keming Biotech Co. Ltd, Jiangsu, China). For the H_2_O_2_ assay, leaves (0.1 g) were homogenized in 1 ml of reagent 1 under cold conditions. The homogenate was centrifuged at 8000 *g* for 10 min at 4°C. For O_2_^−^, 100 mg of frozen leaves was homogenized in 1 ml of extraction solution and, the mixture was then centrifuged at 10 000 *g* for 20 min at 4°C. The absorbance values of the supernatants were determined at 415 nm for H_2_O_2_ and 530 nm for O_2_^−^.

### Determination of malondialdehyde concentration

Frozen leaves (0.1 g) were homogenized in 2 ml of 10% (v/v) TCA solution. The mixture was then centrifuged at 4000 *g* for 10 min. The supernatant was adjusted to a volume of 10 ml with precooled TCA for spectrophotometric analysis of MDA concentration, following a previously established protocol [55].

### Determination of proline concentration

Leaf samples (0.5 g) were homogenized in a 3% (w/v) solution of sulfosalicylic acid, followed by filtration through filter paper [56]. The resulting homogenate was heated at 100°C for 1 h in a water bath after the addition of acid ninhydrin and glacial acetic acid. The reaction was terminated by placing the mixture in an ice bath. Then, the mixture was extracted with toluene, and the absorbance of the fraction aspirated from the liquid phase with toluene was measured at 520 nm.

### Determination of endogenous hormones

*In vitro*-grafted rootstocks and scions were harvested after 5% PEG8000 treatment for 18 days. The hormones GA_1_, GA_3_, GA_4_, GA_7_, and ABA were determined by Nanjing Webiolotech Biotechnology Co., Ltd. Freeze-dried samples (0.8 g) were powdered and extracted with 8 ml of acetonitrile solution. The extraction was centrifuged at 12 000 *g* for 5 min. Subsequently, all supernatants were pooled, and 35 mg of C18 was added for impurity purification. After centrifugation under the same conditions as described above, the supernatant was dried with nitrogen gas. The samples were resuspended with 200 μl methanol and filtered using a 0.22-mm organic phase filter. The hormones were measured by an ESI–HPLC–MS/MS system. HPLC conditions were as follows: the column used was a Poroshell 120 SB-C18 reversed-phase column with a temperature maintained at 30°C; the mobile phase consisted of A:B = (methanol/0.1% formic acid):(water/0.1% formic acid); the flow rate was set at 0.3 ml/min; and the injection volume was 2 μl. Mass spectrometry parameters included ionization mode (ESI positive and negative ion mode monitoring), scan type (MRM), air curtain gas pressure (15 psi), spray voltage (+4000 V), atomizing gas pressure (65 psi), auxiliary gas pressure (70 psi), and atomization temperature (400°C).

### Reactive oxygen species content of guard cells analysis

Guard cell ROS levels were evaluated using H_2_DCF-DA staining [52]. Leaves were submerged in a staining buffer containing 10 mM Tris–HCl, 50 mM KCl, 50 μM H2DCF-DA, and 0.02% Tween-20 at pH 7.2 for 2 h at 25°C in darkness. After removing the excess dye from the leaves with distilled water, fluorescence was observed using a confocal microscope (LSM880, Zeiss, Germany).

### Scanning electronic microscopy

The graft union morphology was analyzed using a scanning electron microscope (SEM; FEI Nova Nano 450, USA) equipped with energy-dispersive spectroscopy. Samples were fixed with 2.5% glutaraldehyde for ~2 h and then dehydrated with graded ethanol/water solutions (50, 70, 90, and 100%). After critical point drying with a Leica EM CPD300, the samples were mounted on SEM stubs and coated with a 10-nm layer of platinum/palladium alloy using a Quorum Q150T ES Plus. SEM imaging was conducted at 5.0 kV.

### Sequencing of sRNA and analysis of sRNA pools

sRNA was isolated using the miRNeasy Mini Kit (Qiagen). sRNA libraries were constructed using the NEBNext Small RNA Library Prep Set (E7330L). BGI (Shenzhen) performed single-end 50-bp sRNA sequencing on the DNBSEQ platform. The SOAPnuke1.5.0 sRNAfilter was used to filter raw reads [57]. After filtering, the clean tags were mapped to the reference genome, miRbase (V22), and other sRNA databases with Bowtie2 [58]. Particularly, cmsearch [59] was performed for Rfam mapping. Since the hpRNA in the RNAi vector corresponds to part of the *JrGA20ox1* sequence, *JrGA20ox1* was used as a reference to map the sRNA reads. Only reads showing 100% identity to the *JrGA20ox1* sequence were preserved for further analysis.

### Transcriptome sequencing and data processing

For transcriptome sequencing, total RNAs were isolated from scions using an RNA extraction Kit (DP432, Tiangen, China). The poly(A) mRNA was enriched and subsequently fragmented randomly. The sequencing adapters were ligated to the generated double-stranded cDNAs. cDNAs with length ~200 bp were screened, amplified, and purified. Transcriptome sequencing was conducted using the Illumina HiSeq™ 2000 system. Paired-end reads were *de novo* assembled with Trinity v2.4.0. The transcripts were annotated using the NR, GO, KEGG, eggNOG and Swiss-Prot databases with an E-value ≤10^−5^. Gene expression levels were quantified using the RPKM (reads per kilobase per million reads) method.

### Dual-luciferase assay

The coding sequences of transcription factors and the promoter sequences were connected to the pGreenII 62-SK and pGreenII 0800-LUC vectors, respectively. These recombinant vectors were transformed separately into the *A. tumefaciens* strain GV3101 (pSoup). The concentration ratio of bacteria used for the injection was 1:10 for pGreenII 0800-LUC:pGreenII 62-SK. The paired plasmids were subsequently co-expressed in young tobacco leaves. A dual-luciferase assay kit (Promega) was employed to assess the activity of LUC and REN luciferases. Results were determined by the ratio of LUC to REN.

## Supplementary Material

Web_Material_uhae143

## Data Availability

All the data supporting the findings of this study are available in the paper and supplementary data.
