# Supplementary material for: JrGA20ox1-transformed rootstocks deliver drought response signals to wild-type scions in grafted walnut
Source: Hortic Res. 2024 May 24;11(7):uhae143. doi: 10.1093/hr/uhae143 (PMC11233861; doi:10.1093/hr/uhae143)
Supplement: Web_Material_uhae143 [file web_material_uhae143.zip › graft-HR_SuppFigure.docx]

**Figure S1.** Average internode length of 2-week-cultured WT, JrGA20ox1-RNAi and JrGA20ox1-OE plants. Statistically significant differences in comparison with WT are indicated (**P* < 0.05, ***P* < 0.05). Data are means ± SD of three biological samples.

**Figure S2.** The effects of *JrGA20ox1* transformation on multiple physiological indexes. (a and b) Contents of chlorophyll (a) and proline (b) in WT, JrGA20ox1-RNAi-1 and JrGA20ox1-OE-1 plants under normal growth condition for 0 (control), 1, 2, 3 and 4 weeks. (c-e) Activities of CAT (c), SOD (d) and POD (e) in WT, JrGA20ox1-RNAi-1 and JrGA20ox1-OE-1 plants normal growth condition for 0 (control), 1, 2, 3 and 4 weeks. (f-h) Contents of MDA (f), O_2_^-^ (g) and H_2_O_2_ (h) in WT, JrGA20ox1-RNAi-1 and JrGA20ox1-OE-1 plants under normal growth condition for 0 (control), 1, 2, 3 and 4 weeks. Different letters indicate significant differences (*P* < 0.05) using one-way ANOVA with Tukey’s test for multiple comparisons. Data are means ± SD of three biological samples.

**Figure S3.** *JrGA20ox1* negatively regulates walnut natural drought tolerance. (a) The phenotypes of WT, JrGA20ox1-RNAi and JrGA20ox1-OE plants under natural drought stress. (b and c) Contents of chlorophyll (b) and proline (c) in WT, JrGA20ox1-RNAi-1 and JrGA20ox1-OE-1 plants under natural drought stress for 0, 2, 4 and 6 hours. (d-f) Activities of CAT (d), SOD (e) and POD (f) in WT, JrGA20ox1-RNAi-1 and JrGA20ox1-OE-1 plants under natural drought stress for 0, 2, 4 and 6 hours. (g-i) Contents of MDA (g), O_2_^-^ (h) and H_2_O_2_ (i) in WT, JrGA20ox1-RNAi-1 and JrGA20ox1-OE-1 plants under natural drought stress for 0, 2, 4 and 6 hours. Different letters indicate significant differences (*P* < 0.05) using one-way ANOVA with Tukey’s test for multiple comparisons. Data are means ± SD of three biological samples

**Figure S4.** The effect of *JrGA20ox1* transformed rootstocks on multiple physiological indexes of nontransgenic walnut scions. (a and b) Contents of chlorophyll (a) and proline (b) in scions of WT/WT, WT/RNAi and WT/OE lines under normal growth condition (control) for 0 and 2 weeks. (c-e) Activities of CAT (c), SOD (d) and POD (e) in scions of WT/WT, WT/RNAi and WT/OE lines under normal growth condition (control) for 0 and 2 weeks. (f-h) Contents of MDA (f), O_2_^-^ (g) and H_2_O_2_ (h) in scions of WT/WT, WT/RNAi and WT/OE lines under normal growth condition (control) for 0 and 2 weeks. Different letters indicate significant differences (*P* < 0.05) using one-way ANOVA with Tukey’s test for multiple comparisons. Data are means ± SD of three biological samples.

**Figure S5.** The effect of *JrGA20ox1* transformed rootstocks on nontransgenic walnut scions under natural drought stress. (a) The phenotypes of WT/WT, WT/RNAi and WT/OE lines under natural drought stress for 0 and 5 hours. (b and c) The contents of chlorophyll (b) and proline (c) in scions of WT/WT, WT/RNAi and WT/OE lines under natural drought stress for 0 and 5 hours. (d-f) The activities of CAT (d), SOD (e) and POD (f) in scions of WT/WT, WT/RNAi and WT/OE lines under natural drought stress for 0 and 5 hours. (g-i) The contents of MDA (g), O_2_^-^ (h) and H_2_O_2_ (i) in scions of WT/WT, WT/RNAi and WT/OE lines under natural drought stress for 0 and 5 hours. Different letters indicate significant differences (*P* < 0.05) using one-way ANOVA with Tukey’s test for multiple comparisons. Data are means ± SD of three biological samples.

**Figure S6.** Dual luciferase assay reveals two transcription factors LOL1 (TF2) and FAR1(TF3) from Fig. 5d could regulate the transcription of multiple genes of Fig. 5d.

**Figure S7.** Phenotypes of stomatal aperture. (a) Phenotypes of stomatal aperture of WT, JrGA20ox1-RNAi (RNAi) and JrGA20ox1-OE (OE) lines under 0% (control) and 5% PEG treatments for 1 week. (b) Phenotypes of stomatal aperture of scions leaves from WT/WT, WT/RNAi and WT/OE lines under 0% (control) and 5% PEG treatments for 1 week.

**Figure S8.** Transpiration rates of WT, JrGA20ox1-RNAi (RNAi, JrGA20ox1-OE (OE), WT/WT, WT/RNAi and WT/OE lines.
